# *Aspergillus tubingensis* Endocarditis: A Case Report and Review of the Literature

**DOI:** 10.1007/s11046-022-00621-0

**Published:** 2022-03-10

**Authors:** Tristan Born, Marion Aruanno, Eleftheria Kampouri, Matteo Mombelli, Pierre Monney, Piergiorgio Tozzi, Frederic Lamoth

**Affiliations:** 1 Infectious Diseases Service, Department of Medicine, Lausanne University Hospital and University of Lausanne, Centre Hospitalier Universitaire Vaudois, Lausanne, Switzerland; 2 Institute of Microbiology, Department of Laboratories, Lausanne University Hospital and University of Lausanne, Rue du Bugnon 46, 1011 Lausanne, Switzerland; 3 Service of Internal Medicine, Department of Medicine, Lausanne University Hospital and University of Lausanne, Lausanne, Switzerland; 4 Cardiology Service, Lausanne University Hospital and University of Lausanne, Lausanne, Switzerland; 5 Cardiac Surgery Division, Lausanne University Hospital and University of Lausanne, Lausanne, Switzerland

**Keywords:** *Aspergillus niger*, Section *Nigri*, Aortitis, Invasive aspergillosis, Fungal biofilm

## Abstract

*Aspergillus* endocarditis is a rare infection that may affect immunocompetent patients following heart valve replacement or heart surgery. We report the case of a 39 year old woman with a history of intravenous drug use who developed endocarditis with direct examination of the resected valve and vegetation showing the presence of mycelia. Cultures were positive for an *Aspergillus* of section *Nigri*, which was subsequently identified as *Aspergillus tubingensis* by sequencing. The clinical course was favorable following surgery and prolonged antifungal therapy (8 months in total). Antifungal susceptibility testing showed good in vitro activity of amphotericin B, voriconazole and echinocandins against planktonic cells of this *A. tubingensis* isolate. However, only amphotericin B displayed significant activity against biofilms. In vitro combinations of voriconazole or amphotericin B with echinocandins did not meet the criteria of synergism. Our review of the literature identified 17 other cases of endocarditis attributed to *Aspergillus* of section *Nigri* with an overall mortality rate of 57% (100% in the absence of surgery). Endocarditis caused by *Aspergillus niger* and related cryptic species are rare events, for which surgical management appears to be crucial for outcome. While amphotericin B was the only antifungal drug displaying significant anti-biofilm activity, the type and duration of antifungal therapy remain to be determined.

## Background

*Aspergillus* spp. are opportunistic mold pathogens causing invasive aspergillosis in patients with severe immunosuppression, such as hematologic cancer patients or transplant recipients [1]. While the lung represents the main port of entry, primary extra-pulmonary infections are occasionally observed. *Aspergillus* endocarditis is a very rare entity, which has been reported not only in immunocompromised patients, but also in immunocompetent individuals with a history of valve replacement, open heart surgery, or intravenous drug use [2–4]. In this setting, direct inoculation of the fungus in blood may occur via contaminated material. *Aspergillus* endocarditis is notoriously difficult to treat in the absence of evidence-based recommendations regarding the type and duration of antifungal therapy [3]. Valve replacement is considered to be mandatory [3]. The mortality rate is very high and there is an important risk of relapsing infection among survivors, which usually poses the indication for prolonged suppressive therapy [3].

While *Aspergillus fumigatus* represents the major cause of *Aspergillus* endocarditis and invasive aspergillosis in general, *Aspergillus niger* and other *Aspergillus* spp. of section *Nigri* account for some cases [5–18]. Indeed, *Aspergillus niger* is ubiquitous in the environment and has been recognized as a relatively frequent contaminant of hospital indoor environment (e.g. following building renovation works) or infusion fluids (e.g. peritoneal dialysate, dextrose infusion) [19–22]. Among *Aspergillus* species of section *Nigri*, cryptic species are frequently recovered in clinical specimens with *Aspergillus tubingensis* accounting for about 20% to over 50% of them [23–27]. This pathogen has been associated with decreased azole susceptibility in some cases [24–26].

We report here a case of *A. tubingensis* prosthetic valve endocarditis in an apparently immunocompetent patient known for intravenous drug use, which was successfully treated with surgery and combined antifungal therapy. This work was completed by a review of the literature of *Aspergillus* section *Nigri* endocarditis and by in vitro experiments of anti-planktonic and anti-biofilm activity of the different antifungal drugs against the isolated pathogen.

## Case Presentation

**Fig. 1:**
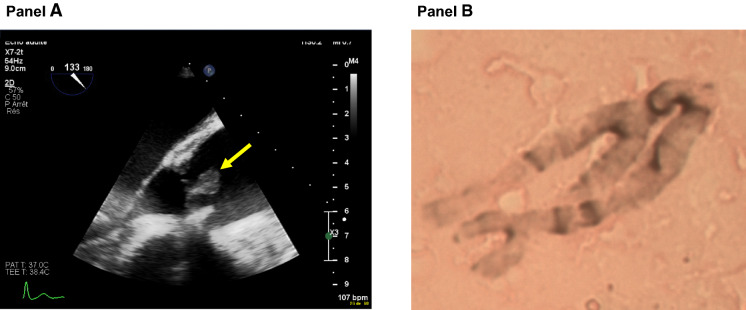
Case report: radiological and microbiological images. **Panel A** Image of the transoesophageal cardiac echography showing a motile element of 11 mm (yellow arrow) on the right coronary leaflet of the biological prosthetic aortic valve. **Panel B** Image of the direct examination of the valvular tissue after silver staining showing mycelial elements with septate and 45° branching hyphae consistent with an *Aspergillus* spp

A 39 year-old woman known for active intravenous drug use (mainly cocaine) and a biological aortic valve replacement for *Enterococcus faecalis* endocarditis more than 10 years ago was admitted to the emergency room for altered level of consciousness and septic shock. Blood cultures drawn at admission were positive for *Proteus mirabilis* (all four bottles from two distinct pairs). Her hemodynamic condition rapidly improved after initiation of broad-spectrum antibiotic therapy and subsequent switch for ceftriaxone and addition of gentamicin after identification of the bacterial blood pathogen. A transoesphageal cardiac ultrasound revealed an 11 mm motile element on the right coronary leaflet of the aortic valve (Fig. 1, panel A). The patient underwent biological replacement of the aortic valve. A piece of the resected valvular tissue and vegetation was sent to the laboratory of microbiology. Direct examination did not reveal the presence of bacteria at gram staining, but thin septate and branched mycelial elements consistent with an *Aspergillus* spp. were visualized at silver staining (Fig. 1, panel B). Conventional cultures of the resected valve did not reveal the presence of bacteria, but a filamentous fungus grew on all plates after 24 h of incubation, which was subsequently identified as an *Aspergillus* of section *Nigri*. The direct panfungal PCR (targeting the 18S rDNA) was positive for an *Aspergillus* of section *Nigri*. Of note, the direct eubacterial PCR (targeting the 16S rDNA) was also positive for *P. mirabilis*. Another piece of the cardiac valve was also sent for histopathological examination, which showed fibrino-leucocytic debris without mycelial elements at Grocott staining.

Antifungal susceptibility testing of the *Aspergillus* section *Nigri* was performed by Sensititre YeastOne™ (Trek Diagnostics Systems, ThermoFisher Scientific, Cleveland, OH, USA) according to manufacturer’s recommendations. Minimal inhibitory concentrations (MIC) were as follow: amphotericin B 1 mg/l, voriconazole 1 mg/l, itraconazole 1 mg/l, posaconazole 0.25 mg/l, caspofungin 0.015 mg/l, anidulafungin < 0.015 mg/l, micafungin < 0.008 mg/l. Although interpretive clinical breakpoints are lacking for *Aspergillus* of section *Nigri*, these values were below the reported epidemiological cut-off values (ECVs) and were considered as indicative to guide antifungal therapy [28–30].

The galactomannan and 1,3-beta-d-glucan were positive in serum (optical density 2.34 and > 500 pg/ml, respectively). The diagnostic work-up was completed by a cerebral and thoraco-abdominal CT-scan that did not show evidence of secondary foci of the infection. A diagnosis of mixed bacterial (*P. mirabilis*) and fungal (*A. niger*) endocarditis secondary to intravenous drug injection was established. Intravenous liposomal amphotericin B (L-AMB, 5 mg/kg/day) was started with addition of caspofungin (70 mg loading dose, followed by 50 mg/day from day 2). Three days later, L-AMB was substituted for intravenous voriconazole (6 mg/kg bid on day 1, followed by 4 mg/kg bid from day 2) and gentamicin was interrupted due to the development of acute renal failure. The clinical and biological course was favorable with serum galactomannan rapidly turning negative and a decline of serum 1,3-beta-d-glucan. Documentation of the clearance of the *P. mirabilis* bacteremia was obtained at day 5 from the start of antibacterial therapy and the patient was treated with ceftriaxone for a total of 6 weeks after surgery. The patient was discharged at post-operative day 10 and was followed at the outpatient clinic for monthly clinical evaluation and laboratory tests (hepatic tests, creatinine, complete blood count). Voriconazole was switched from intravenous to oral administration after 7 days and combination therapy of voriconazole and caspofungin was continued for a total of 14 days. The trough concentration of voriconazole measured at that time (day 14) was 4.5 mg/l. Voriconazole monotherapy was continued for a total of 47 days and was then substituted for oral isavuconazole (200 mg tid on days 1 and 2, followed by 200 mg qd) because of abnormal hepatic tests. The PET-CT performed 6 months after surgery showed no signs of recurrent infection. Isavuconazole was stopped after a total of 8 months of antifungal therapy because of shortage of the drug and multiple interactions with the patient’s psychotropic medications (risperidone, pregabalin, clorazepate, trazodone) precluding administration of other azole drugs. About 6 months later, the patient developed a novel episode of endocarditis, which was suspected to be recurrent *Aspergillus* endocarditis, despite a negative galactomannan in serum. However, microbiological analyses of the resected valve established the diagnosis of *Saccharomyces cerevisiae* endocarditis without any evidence of recurrent *Aspergillus* infection [31].

## Methods

The mold recovered by culture of the infected cardiac valve and identified as an *Aspergillus* of section *Nigri* by morphological examination was further characterized at the species level by beta-tubulin (*BenA*) sequencing using primers previously described [32]. Drug interactions were tested by the checkerboard dilution method using the broth microdilution protocol of the Clinical and Laboratory Standards Institute (CLSI) M38 (3rd edition) for MIC determination [33]. The fractional inhibitory concentration index (FICI) was calculated and interpreted as previously described [34]. In order to test the antifungal activity against the *Aspergillus* biofilms, we measured the reduction of the tetrazolium salt 2,3-bis(2-methoxy-4-nitro-5-[(sulphenylamino)carbonyl]-2H-tetrazolium-hydroxide (XTT) by metabolic active fungal cells within the biofilm, as previously described [35, 36]. In brief, from a suspension of 2.10^3^ conidia/mL in MOPS-buffered RPMI 1640 (Sigma-Aldrich, Saint-Louis, MO), 200 µL were incubated for 48 h at 37 °C in a 96-well plate (Corning, NY, USA). Biofilms were washed with PBS twice to eliminate non-adherent cells and incubated at 37 °C for 16 h in MOPS-buffered RPMI 1640 with addition of antifungals (amphotericin B, voriconazole or caspofungin, Sigma-Aldrich, Saint-Louis, MO) at different concentrations. Wells were rinsed with PBS twice to eliminate antifungals and incubated with 200 µL of a saline solution containing 200 µg/mL XTT and 25 µM menadione (Sigma-Aldrich, Saint-Louis, MO) for 2 h at 37 °C in the dark. The color change was measured by the LUMIstarOmega microplate reader (BMG LABTECH, Ortenberg, Germany) using a 485 nm filter. The sessile minimal inhibitory concentration (SMIC) was assessed as the concentration of the drug achieving a 50% decrease of absorbance.

## Results

**Fig. 2:**
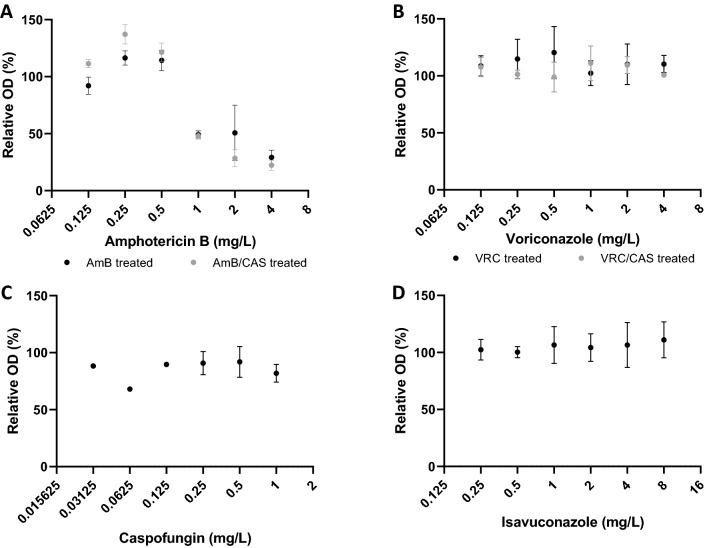
Anti-biofilm activity of antifungal drugs against *Aspergillus tubingensis*. The anti-biofilm activity of antifungal drugs was measured against the sessile forms of the *A. tubingensis* isolate of the present case using the XTT reduction assay. Absorbance (λ_485nm_) was measured after 16 h of drug exposure and expressed as relative optical density (OD). **A** Amphotericin B (AmB) alone and combined with caspofungin (CAS, fixed dose of 0.25 µg/ml), **B** voriconazole (VRC) alone and combined with caspofungin (CAS, fixed dose of 0.25 µg/ml), **C** caspofungin, **D** isavuconazole. The sessile minimal inhibitory concentration (SMIC), defined as the concentration of the drug achieving a 50% decrease of relative OD, was 1 µg/ml, > 4 µg/ml, > 1 µg/ml, and > 8 µg/ml for amphotericin B, voriconazole, caspofungin and isavuconazole, respectively. The addition of caspofungin to amphotericin B or voriconazole did not result in any additive effect

The pathogenic mold was identified as *Aspergillus tubingensis* by sequencing of *BenA* showing a total scores of 898 (100% similitude with *A. tubingensis* voucher IHEM17170 *BenA* gene) and 929 (99% similitude with *A. tubingensis* isolate A87CM *CaM* gene). These sequences as been deposited in GenBank under ID numbers OL771245. In checkerboard dilutions, the interactions of amphotericin B/caspofungin and voriconazole/caspofungin were classified as indifferent with FICIs of 0.75 and 1.125, respectively. Results of the XTT assay are shown in Fig. 2. Only amphotericin B displayed significant activity against sessile cells with a SMIC of 1 µg/ml. Addition of caspofungin to either amphotericin B or voriconazole did not result in enhanced activity.

## Review of the Literature

A systematic search was performed on PubMed (https://pubmed.ncbi.nlm.nih.gov/) using the terms: “endocarditis” or “aortitis” and “*Aspergillus niger*” or “*Aspergillus tubingensis*” or “section Nigri”. Our search identified 17 cases of endocarditis or aortitis caused by *Aspergillus* spp. of section *Nigri*. Description of these cases with addition of the present case report (n = 18) is provided in Table 1. All patients except one had a past history of heart surgery and/or heart valve replacement or valvuloplasty. Only one patient was immunocompromised (acute leukemia). Septic embolisms were commonly observed. Mortality was 100% in the absence or surgical intervention and 45% among patients who underwent surgery of valve replacement and/or thrombus removal. Among the patients who survived without recurrence in follow-up, the duration of antifungal therapy ranged from 6 to 12 months.

**Table 1:** Literature review of endocarditis and aortitis caused by *Aspergillus* species of section *Nigri*

First author, year (reference)	Sex, age, underlying conditions	Site(s) of infection	Mold identification (site, method)	Therapeutic approach	Outcome (duration of follow-up if alive)
Mahvi, 1968 [13]	Male, 9 y.o Aortic valvuloplasty (− 158 days)^a^	Aortic valve, multiple septic emboli	*Aspergillus niger* (aortic thrombus, culture)	No valve surgery (thrombus removal) D-AMB	Death
Moore, 1984 [16]	Male, 63 y.o Aortic valve replacement (biological) (− 53 days)^a^	Prosthetic aortic valve, cerebral embolism	*Aspergillus niger* (valve, culture)	No surgery No AFT (autopsy discovery)	Death
Vivas, 1998 [18]	Male, 44 y.o Aortic valve replacement (mechanical) (− 2.5 months)^a^	Prosthetic aortic valve, popliteal artery embolism	*Aspergillus niger* (valve, culture)	Surgery, D-AMB, then itraconazole (6 months)	Cure, no recurrence (2 years)
Kreiss, 1999 [12]	Male, 57 y.o Mitral valvuloplasty (− 3.5 months)^a^	Mitral valve	*Aspergillus niger* (valve, culture)	Surgery, D-AMB (2 months), then itraconazole (12 months)	Cure, no recurrence (16 months)
Kocazeybek, 2000 [11]	Female, 53 y.o Aortic valve replacement (− 3 months)^a^	Prosthetic aortic valve	*Aspergillus niger* (valve, culture)	Surgery, ABCD, then L-AMB (40 days), then itraconazole (30 days)	Recurrence (after AFT interruption) and death
McCracken, 2003 [15]	Male, 56 y.o Acute myeloid leukemia (no valvulopathy)	Mitral valve, lung, brain, liver	*Aspergillus niger* (valve, blood, liver, PCR)	No surgery, L-AMB	Death
El-Hamamsy, 2004 [9]	Male, 50 y.o Aortic valve replacement (mechanical) (− 9 weeks)^a^	Prosthetic aortic valve	*Aspergillus niger* (valve, culture)	Surgery, AMB and itraconazole	Recurrence and death
Duygu, 2006 [8]	Female, 50 y.o Aortic valve replacement (mechanical) (− 2 months)^a^	Prosthetic aortic valve, ascending aorta (pseudoaneurysm)	*Aspergillus niger* (blood, culture)	Surgery, L-AMB (2 weeks), then itraconazole (6 months)	Cure, no recurrence (24 months)
Badiee, 2009 [6]	Male, 64 y.o Aortic valve replacement (7 months)^a^	Aortic valve	*Aspergillus niger* (valve, culture and PCR, serum)	Surgery, voriconazole	Alive (not specified)
Badiee, 2009 [6]	Female, 35 y.o Aortic valve replacement (− 8 months)^a^	Aortic valve	*Aspergillus niger* (valve, culture and PCR, serum)	Surgery, AMB	Death
Jamieson, 2011 [10]	Male, 67 y.o Aortic valve replacement (biological) and aortoplasty (− 4 months)^a^	Aortic root (pseudoaneurysm), abdominal aorta embolism	*Aspergillus niger* (culture, phenotypic identification)	Embolectomy, voriconazole	Death
Noordally, 2011 [17]	Female, 71 y.o Aortic valve replacement and coronary artery bypass graft surgery (− 9 months)^a^	Ascending aorta, femoral artery embolism	*Aspergillus niger* (thrombus, culture)	Surgery, AMB	Death
Badiee, 2014 [7]	Female, 27 y.o Previous surgery (not specified)	Valve (not specified),	*Aspergillus niger* (valve, culture, and blood, PCR)	Not specified	Not specified
Badiee, 2014 [7]	Female, 67 y.o Previous surgery (not specified)	Prosthetic valve	*Aspergillus niger* (valve, culture, and blood, PCR)	Not specified	Not specified
Badiee, 2014 [7]	Male, 19 y.o Previous surgery (not specified)	Valve (not specified), pulmonary infarct	*Aspergillus niger* (valve, culture, and blood, PCR)	Not specified	Not specified
Arnáiz-García, 2019 [5]	Male, 77 y.o Aortic valve replacement (biological) (− 6 months)^a^	Prosthetic aortic valve	*Aspergillus niger* (valve, culture)	Surgery (AFT not specified)	Not specified
Marro, 2020 [14]	Male, 38 y.o Aortic valve replacement (mechanical) Ascending aorta replacement (− 7 months)^a^	Prosthetic aortic valve, ascending aorta, distal leg and spleen embolisms	*Aspergillus niger* (thrombus, culture)	Surgery, caspofungin then L-AMB (45 days), then voriconazole (6 months)	Cure, no recurrence (6 months)
Present case, 2021	Female, 39 y.o Aortic valve replacement (biological) (− 10 years) Intravenous drug use	Prosthetic aortic valve,	*Aspergillus tubingensis* (valve, culture and PCR sequencing)	Surgery L-AMB (3 days), then voriconazole and caspofungin (14 days), then voriconazole (33 days), then isavuconazole (200 days)	Cure, no recurrence (14 months)

*AFT* antifungal therapy, *AMB* amphotericin B (type of formulation not specified), *D-AMB* deoxycholate amphotericin B, *ABCD* amphotericin B colloidal dispersion, *L-AMB* liposomal amphotericin B

^a^Timing of surgery related to the diagnosis of fungal endocarditis (or admission)

## Discussion and Conclusions

We reported the case of a patient who developed *A. tubingensis* endocarditis on a biological prosthetic valve, probably following direct inoculation of the pathogen in the blood via a contaminated drug injection, in the absence of other primary focus of infection. The patient was successfully treated by surgery and 8 months of antifungal therapy consisting of initial L-AMB therapy for 3 days followed by a combination of voriconazole and caspofungin for 14 days and then voriconazole and isavuconazole monotherapy.

*Aspergillus* endocarditis is a rare event and represents the second cause of fungal endocarditis after *Candida* spp. [37, 38]. Prosthetic heart valves, structural heart disease or past history of endocarditis were the most frequent underlying conditions [37, 38]. History of active intravenous drug abuse has also been reported [37, 39], although it is unclear whether the fungus originates from the drug or its adjuvant, or from the contaminated material of injection.

Our review of the literature identified 17 case reports of endocarditis caused by *Aspergillus* spp. of section *Nigri*. Most of these cases were attributed to *A. niger* by phenotypic identification, but were not characterized at the species level by molecular analysis. Our case represents the first report of endocarditis attributed to *A. tubingensis*, which is actually one of the most frequent cryptic species of section *Nigri* in Europe [24, 25, 27]. This species may exhibit some decrease of azole susceptibility compared to *A. niger *sensu stricto, according to previous in vitro studies [24–26]. Our strain had a voriconazole MIC of 1 mg/l, which actually does not exceed the epidemiological cut-off value (i.e. 2 mg/l) of *A. niger* [29]. Data about the in vitro interactions of voriconazole with caspofungin (or other echinocandins) against *Aspergillus* spp. are controversial and suggest an occasional synergistic effect, which may be strain-dependent [40–42]. Data for *A. niger* are scarce, but the interaction was classified as indifferent for a majority of isolates [41]. Similar observations have been reported for the interaction of amphotericin B and caspofungin against *Aspergillus* spp. and *A. niger* [43]. Our checkerboard testing with the present *A. tubingensis* strain showing indifferent interactions for these antifungal combinations are consistent with these previous observations. Data on antifungal activity on biofilms are more scarce. One study assessed the individual and combined effect of voriconazole, amphotericin B and caspofungin against 22 *Aspergillus* spp. including 3. *A. niger* [44]. Our results are comparable to their observations with only amphotericin B displaying significant in vitro activity against the biofilm of *A. tubingensis* (SMIC 1 mg/l). However, contrarily to the results of Liu et al. [44], we did not observe any enhanced activity with the addition of caspofungin at therapeutic concentrations to either amphotericin B or voriconazole.

While the optimal antifungal therapy of *Aspergillus* endocarditis remains to be determined, our review of the literature shows that surgery is a key determinant of therapeutic success, which is in accordance with experts’ recommendations [3, 4]. The crucial role of surgery is not surprising as secondary embolic events represent a very frequent complication and the major cause of death among these patients. Fortunately, our patient did not experience any symptom or radiological evidence of thrombo-embolic complications before surgery, which was probably a good prognostic factor.

Another debated question in the management of *Aspergillus* endocarditis is the duration of antifungal therapy. In our literature review, all patients who survived without recurrence received antifungal therapy for at least 6 months. Whether this duration can be shortened is unknown. The use of PET-CT in follow-up may help in determining the duration of treatment as it was the case here.

In conclusion, this case of *A. tubingensis* endocarditis is the first one formally attributed to this cryptic species and among the rare ones attributed to *Aspergillus* of section *Nigri* that have been reported until now. Our in vitro analyses suggest that amphotericin B is the only antifungal drug displaying significant anti-biofilm activity, which may support its use as first-line therapy. We did not observe any benefit of the addition of an echinocandin on the basis of our in vitro data for the present case, but clinical data are lacking to determine the optimal antifungal regimen for such rare infections.

## Data Availability

The dataset of this case report is available upon reasonable request of the editors and with respect of the confidential rules of our institution and anonymity of the patient.
